# Carbapenem-resistant Citrobacter amalonaticus and VRE bacteraemia in an immunocompetent patient after a urological Rezum procedure

**DOI:** 10.1099/acmi.0.000852.v4

**Published:** 2024-10-10

**Authors:** David Midlick, Jason Harhay, Nathan A. Summers

**Affiliations:** 1 College of Medicine, University of Tennessee Health Science Center, Memphis, TN, USA; 2 Department of Medicine, Division of Infectious Diseases, University of Tennessee Health Science Center, Memphis, TN, USA

**Keywords:** bacteraemia, carbapenem-resistant Enterobacterales, *Citrobacter amalonaticus*, Rezum

## Abstract

In this report, we discuss the case of a 62-year-old man who presented with gross haematuria, fever, and chills 1 day after undergoing a Rezum procedure and was found to have carbapenem-resistant *Citrobacter amalonaticus* and vancomycin-resistant *Enterococcus faecalis* bacteraemia. The patient was treated with daptomycin, eravacycline, and ceftalozane–tazobactam with positive results. We discuss our case and treatment of *C. amalonaticus* bacteraemia, a pathogen with limited existing literature on its incidence, presentation, and treatment.

## Data Summary

No data were generated or reused.

## Introduction

*Citrobacter amalonaticus* is a Gram-negative bacillus of the family *Enterobacteriaceae* first described in 1971 that, like other members of its genus, is considered to be an opportunistic pathogen in nosocomial settings [1,2]. The literature on *C. amalonaticus* is limited, but it has been associated with urinary tract infection (UTI) and sepsis in humans. On the other hand, the growing prevalence of carbapenem-resistant-Enterobacterales (CRE) infections in healthcare settings has been attracting widespread attention. Here we present the case of an immunocompetent patient who was found to have *C. amalonaticus* and vancomycin-resistant *Enterococcus faecalis* (VRE) bacteraemia after undergoing a urological Rezum procedure. The treatment of both organisms is detailed here, but our focus for this report is on the *C. amalonaticus*. The case report is meant to highlight a case of carbapenem-resistant Enterobacterales after a Rezum procedure, which we believe is this first to be documented, and outline a treatment regimen that could be used in similar presentations elsewhere.

## Case presentation

A 62-year-old African-American male with a past medical history of intellectual disability, epilepsy, chronic urine retention managed with a Foley catheter, hypertension and hypothyroidism presented with a 12 h history of haematuria, fever, and chills.

One day prior to presentation, the patient underwent an elective Rezum procedure. A Rezum procedure (also called water vapour thermal therapy) is a minimally invasive intervention for benign prostatic hyperplasia where bursts of steam are delivered into the prostate with a transurethral cystoscope device to induce cell necrosis and shrink the prostate [3]. The procedure was uncomplicated, and the patient went home the same day. That evening, the patient developed gross haematuria with fever and was brought to the emergency department (ED).

At presentation, the patient was febrile to 39.3 °C, tachycardic to 110 beats per minute but otherwise haemodynamically stable. White blood cell count (WBC) was 2.1×10^3^ cells mm^−3^. Urology was consulted in the ED and attempted Foley irrigation with relief of 100 ml of clot burden, but dark haematuria persisted. Urology began continuous bladder irrigation with glycine suspension. Medicine was consulted due to concerns over sepsis and the patient was started on ceftriaxone empirically for presumed UTI.

Blood cultures originally resulted in Gram-negative rods and Gram-positive cocci in chains and the antibiotics were changed to vancomycin and piperacillin–tazobactam. Urine cultures were negative. On the second day of admission, blood cultures grew *C. amalonaticus* and vancomycin-resistant *E. faecalis*, both identified by MALDI-TOF. As seen in Table 1, the *Citrobacter* was noted to be resistant to meropenem, ertapenem and ciprofloxacin, with concerning results for piperacillin–tazobactam (sensitive but MIC of 16/4 mg l^−1^) but good sensitivity to ceftalozane–tazobactam (MIC <0.25 mg l^−1^). At this time, infectious disease was consulted, and the antibiotics were changed to daptomycin 10 mg kg^−1^ daily, eravacycline 1 mg kg^−1^ every 12 h and ceftalozane–tazobactam 3 gm every 8 h.

**Table 1.** Resistance Values*

	*C.amalonaticus*		Vancomycin-resistant *E. faecalis*	
Antibiotic	Interpretation	MIC dilution (mg l^−1^)	Interpretation	MIC dilution (mg l^−1^)
Amikacin	S	≤8		
Amoxicillin–clavulanate	I	16/8		
Ampicillin	R	>16	S	4
Ampicillin–sulbactam	R	>16/8		
Aztreonam	R	>16		
Cefazolin	R	>16		
Cefepime	R	16		
Cefiderocol†				
Cefoxitin	S	8		
Ceftazidime	R	>16		
Ceftazidime–avibactam	R	>8/4		
Ceftolozane–tazobactam	S	≤1/4		
Ceftriaxone	R	>32		
Cefuroxime	R	>16		
Ciprofloxacin	R	2		
Daptomycin			S	2
Ertapenem	R	>2		
Gentamicin	S	≤2		
Gentamicin synergy			S	
Linezolid			S	2
Meropenem	R	4		
Meropenem–vaborbactam	I	8		
Moxifloxacin	I	4		
Penicillin			S	8
Piperacillin–tazobactam	S	16/4		
Streptomycin synergy			S	≤1000
Tetracycline	R	>8		
Tigecycline	S	2		
Trimethoprim–sulfamethoxazole	R	>2/38		
Vancomycin			R	>32

a*All breakpoints were determined according to Clinical and Laboratory Standards Institute (CLSI) guidelines.

b†The report noted that the *Citrobacter* was susceptible to cefiderocol based on Kirby-–Bauer Iinterpretation.

Throughout hospitalization, the patient remained afebrile, normotensive and at his mental baseline. Repeat blood cultures on days 4 and 6 of admission were negative. A midline catheter was placed on day 7 and the patient was discharged on day 7 to a skilled nursing facility (SNF) to complete a 14 day regimen of ceftalozane–tazobactam and daptomycin. The eravacycline was discontinued on transfer to the SNF. Subsequent testing at the Tennessee Department of Health after patient discharge confirmed carbapenem resistance and this *Citrobacter* isolate was specifically negative for NDM, VIM, IMP, KPC, and OXA-48 expression using Streck ARM-D RT-PCR testing.

Further review of the medical record showed that this patient presented at the hospital emergency department again approximately 1 month later for a breakthrough seizure and a urine culture grew *Citrobacter amalonaticus*. The patient was asymptomatic and did not meet sepsis criteria. Infectious disease was consulted but no antibiotics were given due to the patient’s colonization status. The patient was discharged from the emergency department without admission.

## Discussion

First described in 1971, *C. amalonaticus* has been isolated in human urine, faecal and wound samples, usually in hospitalized or immunocompromised patients; however, this pathogen was thought to be rare in humans [1,4]. The earliest mention of * C. amalonaticus* as a human pathogen was reported in five patients from 1972 to 1978 at the Seattle Veterans Administration Medical Center in the USA, two of whom had a UTI as the primary infection [1,2]. A case review from Marseille, France in 2016 noted 36 cases of *C. amalonaticus* over a 13 year period in hospitalized patients with the majority of isolates coming from urine sample (80%) [1]. *C. amalonaticus* has also been identified in Malawi, PR China, Taiwan, ROC, and Italy [1].

*Citrobacter* spp. in general are opportunistic nosocomial pathogens that comprise 3–6% of all human Enterobacterales infections [5]. While the majority of *Citrobacter* infections are due to *C. koseri* and *C. freundii*, *C. amalonaticus* is a pathogen that has limited published literature on its prevalence, clinical presentation, and treatment [5]. What is worrisome is the prevalence *Citrobacter* spp. among carbapenem-resistant Enterobacterales, a growing worldwide health concern. *Citrobacter* as a genus has been shown to develop resistance to carbapenems via plasmid-encoded carbapenemases, including acquisition of metallo-beta-lactamases and OXA carbapenemases [5,6]. In 2019, a urine isolate of *C. amalonaticus* demonstrated carbapenem and colistin resistance conferred by plasmid-encoded enzymes [5].

In contrast to general trends in *Citrobacter* infections, our patient was not immunocompromised. He was HIV-negative and had not had any recent hospital exposure or prolonged course of steroids. His chronic urinary retention had been managed with a Foley catheter that was in place for approximately 3 months prior to the Rezum procedure. We hypothesize that he may have been colonized with *C. amalonaticus* in his genitourinary tract, potentially secondary to his chronic catheter, and the bacterium was introduced into his bloodstream by the Rezum procedure. Urethral trauma was evident upon presentation, as evidenced by gross haematuria and clot passage with bladder irrigation. This is supported by a positive urine culture of the same *Citrobacter* species taken during another ED visit a month after his initial presentation.

One study noted that the most common complications of Rezum procedure are dysuria, haematuria and UTI, and the need for >50% of patients to require a catheter post-operatively [3]. In a pilot study from 2015 involving 65 patients in the Dominican Republic, the Czech Republic and Sweden, 13.8% (9 of 65) developed haematuria and 20% (13 of 65) developed a UTI post-operatively, but no patients were found to be bacteraemic [7]. The study did not detail the specific organisms that were responsible for the UTIs. A review of the literature did not reveal any cases of bacteraemia in patients undergoing the Rezum procedure and we believe this case is the first of its kind.

Regarding treatment, we opted for a higher dose of daptomycin to cover the VRE based on previous works [8,9]. For the *Citrobacter*, we chose ceftalozane–tazobactam, a novel but proven agent against multidrug-resistant pathogens, including those producing amp-C type beta-lactamases and extended-spectrum beta-lactamases (ESBLs) [10]. It is to be noted that per the 2023 Infectious Diseases Society of America Antimicrobial Resistance guidance, the recommended treatment options for CRE-identified complicated UTI (cUTI) is ceftazidime–avibactam, meropenem–vaborbactam, imipenem–cilastatin–relebactam or cefiderocol [11]. Our isolate of *Citrobacter* was noted to be resistant to three of these four agents and we did not have imipenem–cilastatin–relebactam available in our hospital formulary.

We also chose eravacycline, a relatively new synthetic fluorocycline, to provide additional coverage of both the *Citrobacter* and VRE while awaiting clearance of his bacteraemia and additional susceptibility testing [12]. Eravacycline shares a similar structure and spectrum of antimicrobial coverage with tigecycline, but the former has been shown to have lower MICs versus *Enterobacteriaceae* and is less prone to efflux [13,14]. Of note, specific data on prostatic penetration by eravacycline and tigecycline is limited, but a case study from 2020 demonstrated successful treatment of prostatitis due to multidrug-resistant *Escherichia coli* with tigecycline monotherapy [14]. Our patient was never formally diagnosed with prostatitis but given his Rezum procedure the prostate was likely the source of his bacteraemia.

As mentioned earlier, subsequent testing performed at the Tennessee Department of Health was negative for expression of the five carbapenemases tested; however, no genotypic testing for AmpC or ESBL production was performed. Furthermore, no carbapenem inactivation method was performed on our *Citrobacter* isolate. The lack of these studies does limit the overall evaluation of this specimen in the context of treatment of a multidrug-resistant organism.

In conclusion, we have detailed a rare case of *C. amalonaticus* bacteraemia following a Rezum procedure. The presentation may be nonspecific, but identification with MALDI-TOF appears to be an accurate way to distinguish *C. amalonaticus* infection. If multidrug-resistant *C. amalonaticus* is identified, consultation of infectious disease and timely initiation of effective antibiotics are required to prevent morbidity and mortality in bacteraemic patients.

## Abbreviations

CRE: carbapenem-resistant-Enterobacterales
cUTI: complicated urinary tract infection
ED: emergency department
SNF: skilled nursing facility
UTI: urinary tract infection
VRE: vancomycin-resistant Enterococcus faecalis
WBC: white blood cell count
